# Digital Twin for Human–Robot Interactions by Means of Industry 4.0 Enabling Technologies

**DOI:** 10.3390/s22134950

**Published:** 2022-06-30

**Authors:** Abir Gallala, Atal Anil Kumar, Bassem Hichri, Peter Plapper

**Affiliations:** Department of Engineering, University of Luxembourg, 6, Rue-Kalergi, L-1359 Luxembourg, Luxembourg; atal.kumar@uni.lu (A.A.K.); bassem.hichri@uni.lu (B.H.); peter.plapper@uni.lu (P.P.)

**Keywords:** Industry 4.0, digital twin, human–robot interaction, collaborative robot programming, mixed reality

## Abstract

There has been a rapid increase in the use of collaborative robots in manufacturing industries within the context of Industry 4.0 and smart factories. The existing human–robot interactions, simulations, and robot programming methods do not fit into these fast-paced technological advances as they are time-consuming, require engineering expertise, waste a lot of time in programming and the interaction is not trivial for non-expert operators. To tackle these challenges, we propose a digital twin (DT) approach for human–robot interactions (HRIs) in hybrid teams in this paper. We achieved this using Industry 4.0 enabling technologies, such as mixed reality, the Internet of Things, collaborative robots, and artificial intelligence. We present a use case scenario of the proposed method using Microsoft Hololens 2 and KUKA IIWA collaborative robot. The obtained results indicated that it is possible to achieve efficient human–robot interactions using these advanced technologies, even with operators who have not been trained in programming. The proposed method has further benefits, such as real-time simulation in natural environments and flexible system integration to incorporate new devices (e.g., robots or software capabilities).

## 1. Introduction

Traditional production systems face a variety of difficulties, such as high demand for customized products, just-in-time production, shorter product life cycles, small batch sizes, etc. [1]. Such challenges have led to a new manufacturing trend that is moving toward more hybrid and flexible production systems. Industrial robots play an important role in these hybrid production lines to improve their efficiency and quality. Despite the advances in the field of robotics, the presence of human workers is still required in many tasks due to their superior analytical and dexterous abilities [2]. In many cases, it has been proven that the combination of superior human capabilities with partial automation is more fruitful than fully automated lines that only use industrial robots [3]. As a result, a majority of industries focus on active human–robot interactions (HRIs) to evolve their production lines [4].

With the onset of Industry 4.0, manufacturing industries have increasing access to various technology drivers, such as big data and analytics, the Industrial Internet of Things (IIOT), cyber–physical systems (CPSs), cloud computing, additive manufacturing and augmented, virtual and mixed realities (AR/VR/MR), among others [5]. More recently, the concept of digital twins (DTs) has been introduced alongside these rapidly expanding technologies [6,7]. Digital twins offer a physical model in real space that can be connected with a corresponding virtual model in virtual space using data and information [8]. As a DT resembles its physical twin exactly, it offers users an extremely precise, high-fidelity, and real-time controllable performance [9]. These features are desirable in situations that require safe, efficient, and rapid interactions between the user and the machine, i.e., they are apt for situations that involve HRIs [10]. Since DT-driven HRIs offer the features of fast integration and easy production reconfiguration, they have also become a popular and reliable approach for flexible manufacturing tasks [11,12].

Powerful and intuitive interfaces are needed to achieve secure and able digital twin-guided HRIs [13]. Additionally, these interfaces must favor the well-being of the workers while enhancing their technological acceptance in a forthcoming industrial scenario at the same time [14]. Although some recent works have demonstrated the use of AR-based DT interfaces for HRIs [15,16], there is still a lack of clarity regarding the design and assessment methods.

A diverse range of industrial applications requires the simulation of planned motions. This enables the avoidance of defects and collisions and the optimization of planned tasks before they are executed by real robots.

This work envisioned the future of robot programming and robot simulation within an industrial environment in which humans and robots work side by side. The main objective of this work was to build and demonstrate a new digital twin-based framework that was designed to enhance human–robot interactions, robot programming and real-time simulation in real environments. The proposed approach would be required to create a flexible real-time service-based framework for both vertical and horizontal integration. It would also be needed to provide an intuitive and human-friendly usage for unskilled workers. A horizontal technological architecture based on the Internet of Things, mixed reality, artificial intelligence and system integration was used during the implementation of different steps.

The following contributions are made in this paper:

- A novel digital twin for a human–robot interaction framework using Industry 4.0 enabling technologies;
- A use case study of the use of the proposed framework with an industrial robot in real-time.

The rest of the paper is organized as follows. Section 2 presents the theoretical background that was needed for the work, along with the current state-of-the-art technology, Section 3 presents the proposed framework, Section 4 includes the use case study for the use of the proposed framework with a real robot. Then, the discussions and conclusions are presented in Section 5 and Section 6, respectively.

## 2. Theoretical Background and State-of-the-Art Technology

### 2.1. Human–Robot Interactions for Robot Programming

Interactions between human beings and robots have changed considerably throughout history. They started with simple physical interactions using basic tools, such as a mouse or a keyboard, following which they developed to the use of touch screens as interactive interfaces. Over the past few years, robots have started to easily interact with human partners via gestures or voice, thanks to their growing autonomy and the introduction of new software and hardware capabilities [17]. Gradually, the relationship between robots and humans has changed to resemble more human–human interactions due to the transformation of robots into more sophisticated co-working partners [18]. This evolution has impacted robot programming methods as well. Furthermore, industrial deviations in manufacturing processes to incorporate hybrid teams have increased the need for more efficient interfaces [19].

To program collaborative robots (robots that are intended for direct human–robot interactions within a shared space) for the previously mentioned types of interaction, different programming approaches exist. We can mainly organize them into two categories: online and offline programming [20]. Online programming is further categorized into two types: teach pendant and lead-through programming [4].

**Teach pendant programming** involves a hand-held device, which is generally attached to a robot and used to teach the robot specific points, locations and path sequences. This technique is suited for applications that have a point-to-point control and for which only one controller is sufficient to determine the trajectory of the robot. However, this method has several limitations: (1) it is difficult to achieve high accuracy for several geometrical trajectories; (2) it is difficult to connect to external devices; and (3) it interrupts production lines when a new task is programmed [21].

**Lead through programming**, also known as programming by demonstration (PbD), aims to enable robots learn new skills from human guidance. It helps non-experts to teach robots without any previous programming knowledge as this method just needs the operator to guide the robot manually into the desired position and record the path [22]. However, this technique still cannot be applied to teach complex collaborative tasks.

**Offline robot programming** helps to resolve the limitations of online methods. It generally involves a dedicated programming and simulation software that aims to design and plan robot movements. Commonly, it is the remote simulation of a 3D graphical model of the work cell environment [23].

This method ensures that planned control algorithms can be implemented correctly before their execution on a real robot [24]. It also assists in complex task implementation and the establishment of connections with external devices. Furthermore, it reduces the downtime of the robot. However, it takes a long time to program the simulation before using it on the robot.

**VR-based robot programming** is an offline programming method in which the system guarantees secure and offhand virtual programming [25]. The trajectory planning is executed in the virtual environment by the operator dragging the digital model of the robot to the final desired position in order to simulate the movement. In short, this approach combines the features from both offline and online methods to take advantage of the online programming method using offline software.

**AR-based robot programming** provides excellent opportunities for HRIs and has been widely explored for use in telerobotics. It allows the worker to operate as though the worker were in the remote working environment [26]. Its potential for programming industrial robots has been explored in the review work of Pan [27], in which the author analyzed both online and offline robot programming (OLP) using AR. During the online programming, the worker can move the robot manually to execute simple tasks. However, the robot cannot handle complex tasks. OLP, on the other hand, generates algorithms using 3D models in which the operator can check and simulate the working scenarios, including the safety zones, reachability and movements, before execution using the real robot. OLP is more complex than online programming and requires skilled engineers.

In [28], Fang et al. proposed an interface for HRIs and robot programming that performs a pick and place operation using a virtual robot replica. The use of multiple cameras and a marker cube also enables operators to manipulate the virtual robot.

### 2.2. Digital Twins in Industry 4.0

A digital twin is a digital replica of a real-world physical system, which aims to enhance the accuracy of simulation capabilities [29]. This concept was first introduced by the National Aeronautics and Space Administration (NASA) when they defined it as a digital representation of a physical flying aircraft that uses the best available physical models, sensor data and flight history [30]. The goal of this process is to continuously update the digital representation with data from the real aircraft, which are then used in the simulations of the DT [31]. In recent years, DTs have been used in the field of manufacturing engineering as virtual correspondents of manufacturing systems [32].

The DT aspect allows for real-time optimization and decision-making interventions, based on the simulation of a virtual model in the real world. This technology should not be confused with the notion of the Internet of Things (IoT). For the IoT, we collect data from a physical object and its environment through sensors, while for DTs, data are injected into the virtual objects. Other emerging technologies, such as AR, HMI dashboards and AI, are also needed to enhance physical connectivity and real-time machine outcomes [33].

Many researchers have investigated the topic of DTs within the context of Industry 4.0 and, more specifically, for human–robot interactions [34,35]. A DT framework for human–robot collaboration within the context of collaborative assembly tasks was presented by Malik in [11]. The presented model was composed of physical and virtual environments. The virtual environment was based on a computer simulation, while the physical environment contained real objects (including humans and robots). The system aimed to simulate its behavior using the formulated DT model. Another DT-based virtual learning environment for collaborative assembly tasks was presented in [36]. It aimed to train unskilled employees to collaborate with robots. Furthermore, Wang [37] proposed another DT framework for real-time HRIs using VR. This system enhanced remote collaboration with industrial robots while executing dangerous construction work. A DT model with a virtual interface for industrial robot trajectory planning was proposed in [38]. This system was only compatible with Fanuc robots. It consisted of two components: a virtual model and a physical robot. The user interface was based on VR, so it was completely virtual and aimed to control the virtual model of the robot. Once the motions were generated, the physical robot moved to the desired position. Very similar VR-based DT systems for industrial robot simulation were presented in [39,40]. The benefits of AR-based DT systems and their combinations were discussed in [41].

From the previously cited methods (which are summarized in Table 1), many problems and limitations were found:

- Traditional robot programming methods require highly paid and qualified engineers and a lot of time (online);
- Unskilled workers cannot program robots easily (text-based offline);
- It is not possible to teach complex collaborative tasks using online robot programming;
- Operators cannot preview movement simulation in real time or in a real environment using offline or VR-based methods;
- Traditional robot programming and simulations do not fit in smart factory environments (text-based offline);
- Human–robot interactions in hybrid teams need to be more efficient and human-friendly;
- The integration of new robots or devices impacts the whole system, which then requires reconfiguration (text-based offline).

To fill these gaps, we proposed a DT-based human–robot interaction framework that aims to provide:

- Intuitive robot programming, so that any unskilled worker can program the robot thanks to the human-friendly interface and the autonomous assistance capabilities of the system while estimating the object position and planning motions;
- Realistic simulation, i.e., a simulation that is performed in a real environment with unpredictable real conditions and objects;
- Flexible system integration, so that it is easy to integrate new devices, robots or features thanks to a broker master interface that connects all of the separated elements with all of their diverse interfaces and platforms.

## 3. Digital Twin Framework Description

The proposed system, called DT-HRI, is a DT framework for human–robot interactions, robot programming and real-time simulation in hybrid teams and combines different Industry 4.0 enabling technologies. DT-HRI is essentially a CPS adoption but with additional components and extended services. It has the most powerful functionalities of a CPS: real-time data transmission between the real and virtual components and a robust computational proficiency for the data processing and analytics.

**Figure 1:**
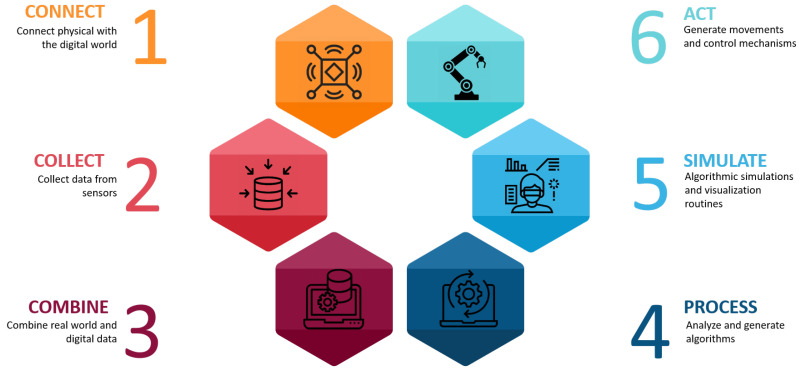
The conceptual digital twin framework.

Figure 1 illustrates the proposed conceptual DT-HRI framework. The system is composed of six steps, each of which is based on at least one key technology. Under the global concept, the system has two main phases: the first, called “3C Setup”, is from steps one to three and the second, called “operate”, is from steps four to six. The first phase is for initiating the environment by including the communication setup, data collection, the merging of real and virtual data and the superimposition of digital contents. The sequence of steps at this level is important and must be followed, while the second phase can be more flexible. The flexibility means that at some levels, the execution can go back and forth, restart the process or skip one or more steps. So, the execution of the first phase is only carried out once at the beginning of the operation, while the second is recurrent and can be repeated as many times as the user needs.

**Step One (“Connect”)**: This consists of the communication establishment layer. It aims to maintain the interconnection bridge between the real world (including machines, devices, sensors and actuators) and the virtual world. This layer enables the exchange of data inside our system. It is important to notice that at this level, both the real and digital worlds should be set up and ready to use, including:

- Machines, devices and sensors that are ready to send and receive data via one of the IoT standard messaging protocols (such as MQTT, DDS, AMQP, etc.);
- Digital content, which includes the digital twins of some of the real components and additional computer-generated data, that is developed, visualized and ready to communicate data.

Since the digital environment is represented in a 3D holographic immersive shape, MR technology is used in the Connect step that enables the visualization and interactions with the digital content. The Connect step is primordial and must always be the first to be initiated before starting any other operation using the DT-HRI system. So, Connect is mainly based on the IIoT, as well as MR technologies.

**Step Two (“Collect”)**: After enabling the Connect step in the different elements of the system, data then need to be collected and transformed into information to synchronize both scenes for the initial start-up of the process. The Collect step is performed using an IoT gateway communication pattern.

**Step Three (“Combine”)**: After connecting the real and digital environments and collecting the data from each part, the Combine step merges both worlds to look as though they are one single environment. In the HRI context, the Combine step has two major goals: (1) the superimposition of the digital twin onto the real-world model, which means that the digital model overlays onto the same position as the physical model; (2) the digital model having the same initial configuration as the physical model. The visualization of holograms in the real environment and interactions with them require MR capabilities. As a result, the Combine step is based on MR technology.

**Step Four (“Process”)**: After setting up all of the environmental components, communication and data collection and the combination of both worlds into one environment, the system is ready to operate. The Process step is for the analytical and processing brain of the system, as the name indicates. At this level, data processing and algorithm generation occurs, based on user behavior and objectives. It has two input resources: the data that are collected from sensors and objects and stored and the data that are received from the intentions and behaviors of the user. As an output, this step translates commands into the specified language of each component and generates motions, trajectories and visual feedback. It requires high computational capabilities, an embedded platform and AI-based functionalities. Meanwhile, it also uses an IoT bridge for internal and external data transmission and exchange.

**Step Five (“Simulate”)**: The algorithms that are generated during the Process step need to be simulated before being transferred to physical devices. Simulation has multiple purposes:

- Simulate the planned behavior and processes;
- Predict errors and prevent problems before they happen;
- Optimize and/or validate the simulated processes;
- Remote assistance, maintenance and monitoring.

It also has many benefits, e.g., in robot programming, the simulation of the movements of a robot before deploying them to the physical robot can increase the iteration time by disclosing hidden outcomes or experimenting with edge movements that may be critical for the physical robot to act directly. Furthermore, the simulation is carried out using MR immersive capabilities. During this step, the user can move around objects so that they can obtain a better preview. In addition, they can interact with objects, send feedback to the system through the immersive interface and validate the processed operations that are to be executed on real physical machines.

**Step Six (“Act”)**: This is the execution of the planned and/or simulated processes by physical devices or machines. The system translates the generated motions into commands that the physical device interfaces can understand. The commands are then transmitted through the IoT bridge.

## 4. Use Case Scenario: Human–Robot Interactions Using Mixed Reality

The proposed DT-HRI method aims to improve upon the current human–robot interactions, robot simulation and robot programming method due to the use of emerging technologies that can be used to fit into smart factory exigencies and demands. For this use case, the system was composed of two pieces of physical equipment: a collaborative robot and an MR head-mounted device (MR-HMD). The physical world consisted of human beings, one or more collaborative robots, an MR-HMD for each user and, when needed, application-related objects (e.g., pieces to assemble or objects to pick and place). The digital world, on the other hand, contained the digital twin model of the robot, additional virtual objects and the user interactive interface (UII). The digital world could recognize and consider human gestures, voices and movements. A third-party engine was responsible for the data collection and processing, along with the generation of the algorithm.

**Figure 2:**
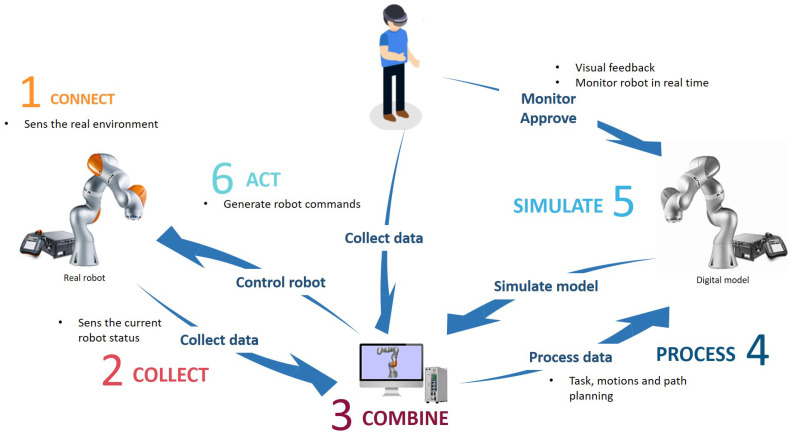
The HRI application of the DT framework.

The HRI-DT framework is illustrated in Figure 2. After connecting the different systems, data were collected from the equipment, sensors and devices, as described in Table 2. After that, a mixed world that recognized the physical robot, related objects and human gestures was created by merging real and virtual worlds, which projected the digital twin, the immersive user interactive interface and a selection of virtual objects.

**Figure 3:**
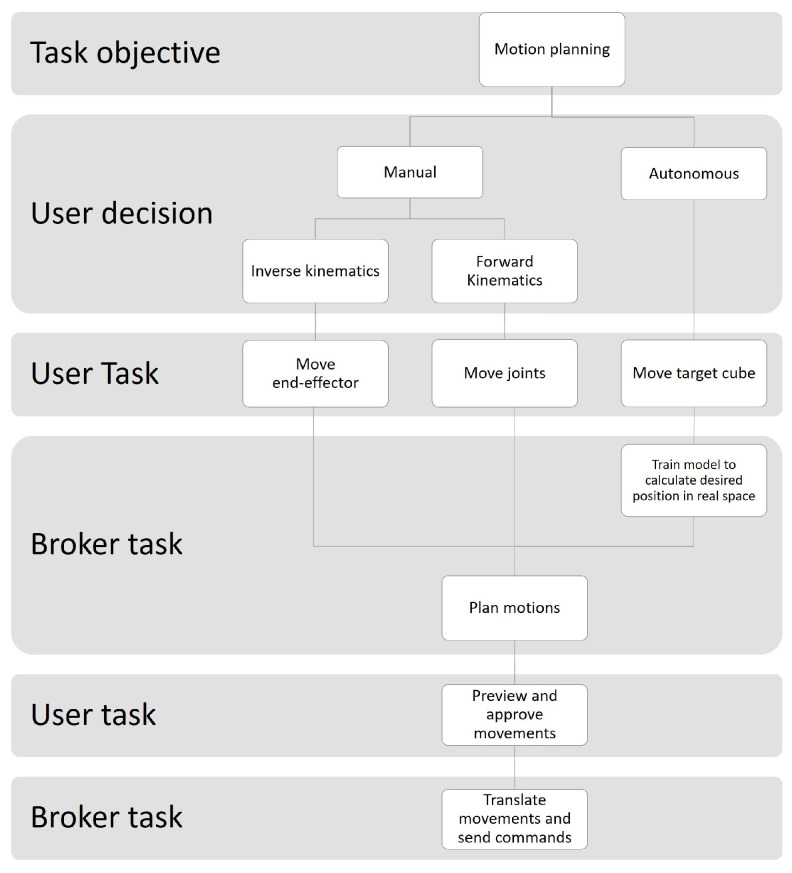
A flowchart of the DT-HRI use case scenario.

The flowchart presented in Figure 3 describes the use case scenario of the robot programming method using the proposed MR-based DT-HRI approach.

In the selected scenario, two types of manipulation were possible:

- Manual Manipulation: The operator used the UII to move the robot arm in the same way as the online programming methods, using either the teach pendant or PbD approach. The difference when using online programming for the proposed DT-HRI approach was that the work was performed on the virtual robot and a simulation preview was available, so the operator could choose to either approve or reject the planned job.
- Autonomous Decision-Making: A broker–processor received the intentions of the user, analyzed the environmental status of both worlds and then generated algorithms for either an action to be taken by a physical entity or feedback to be shown to the user. The decision that was made was first simulated in the digital world before being transmitted to a physical entity after approval.

**Figure 4:**
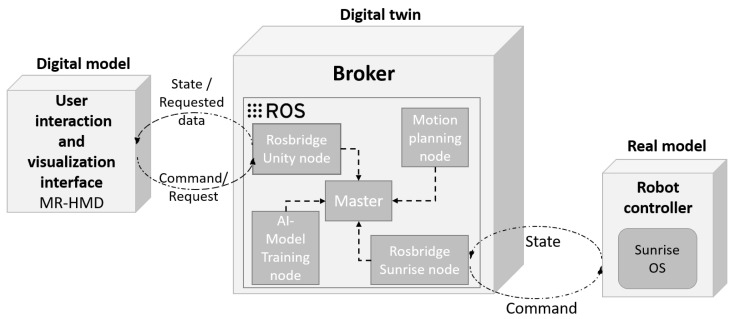
The use case architectural prototype of the DT framework.

Figure 4 presents the use case architectural prototype that was implemented.

The broker had different ROSbridge nodes that were responsible for the IoT communication patterns with different components. The system and its implementation are described in the next paragraph.

### 4.1. ROS-Based Communication Patterns

An ROS communication system is composed of a centralized pattern involving an ROSmaster and multiple ROSbridge nodes. The system has three layers: DT middleware, a physical device and digital device layers.

**Figure 5:**
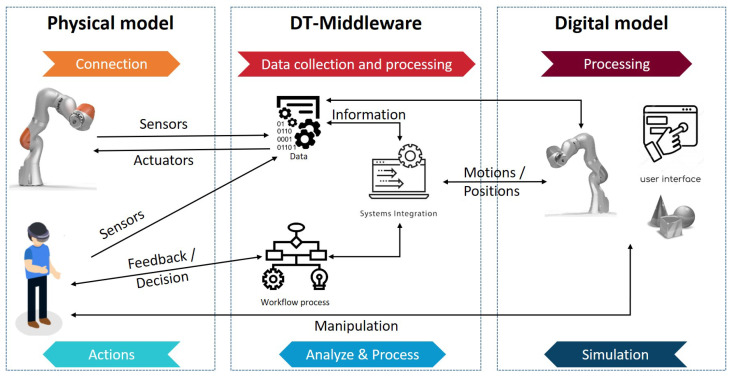
The IoT architectural methodology.

Data that are collected from and delivered to both device layers (the IoT physical and digital device layers are described in Figure 5) pass through the gateway using a publish/subscribe ROS messaging pattern. A physical model is a collaborative robot that has a controller interface that can communicate with the ROS interface. In our use case, we chose the KUKA LBR IIWA. As its name indicates (“IIWA” refers to “intelligent industrial work assistants” and “LBR” refers to “lightweight robot”), the LBR IIWA was designed to work side-by-side with humans and thanks to its integrated sensors, it is flexible, sensitive, safe and independent [42]. The 7-axis LBR IIWA 14 R820 serves as the user’s “third hand”; thus, the interactions between the robot arm and the operator are more efficient and human-like. KUKA’s Sunrise Workbench is a robot controller that uses a publish/subscribe framework to send information about the robot state and receive commands that are to be executed. In addition, the physical device layer of our use case contained an MR head-mounted device (MR-HMD). This device was connected to the internet, so the system was operating through TCP/IP protocol. Table 2 presents the list of sensors in the physical device layer. The robot published the current state of the robot depending on the ROS topic. Topics could be the position of the robot joints, joint velocity, joint torque, cartesian pose, etc. At the same time, it also subscribed to the Command topic to receive new positions or velocity control, either in joint or cartesian space.

On other hand, the ROSsharp was run on the MR-HMD interface in order to establish communication with the Unity ROSbridge in the gateway layer. It published the desired position that was chosen by the user via virtual or real objects and their feedback, such as simulation approval. The MR-HMD–ROSsharp interface subscribed to the State topic as it received the current physical state of the robot or the planned trajectory.

### 4.2. Immersive Mixed Reality Interactive Interfaces

Immersive MR layers merge the real and virtual worlds thanks to the integrated sensors in MR-HMDs, as detailed in Table 2. The chosen MR-HMD in this use case was the Microsoft Hololens 2 device [43].

**Figure 6:**
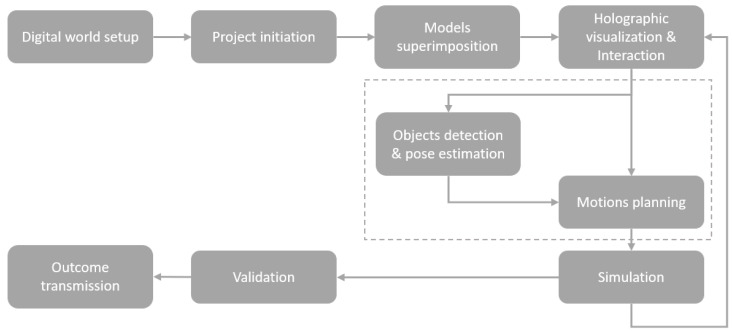
The MR framework phases, from project implementation to execution.

The MR project life cycle, as described in Figure 6, was composed of eight phases and started before the first operation of the system. The system required a one-time pre-usage implementation for the digital world setup.

**Figure 7:**
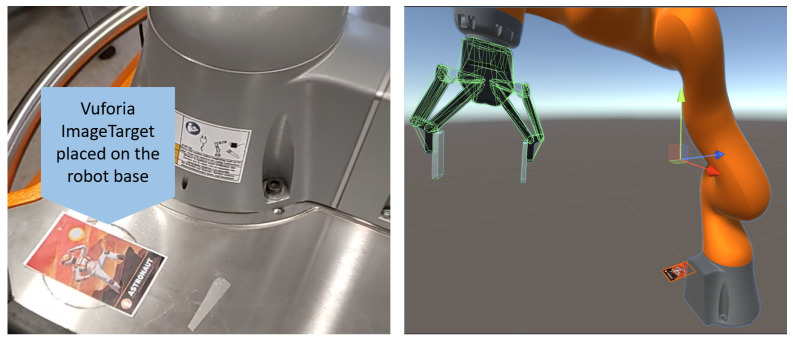
The Vuforia image target for project initiation and model superimposition.

1. Digital World Setup: This phase represented the implementation phase, in which all digital objects were constructed and included in the programming software. A Unity game engine platform was used to implement the MR interface using the C# programming language and a .NET framework. A unified robot description format (URDF) file of each mechanical object was imported into the platform. The URDF XML format described the structural illustration of the KUKA IIWA robot and the attached 2F gripper. Furthermore, the same URDF description was used by ROS to communicate between the physical and digital objects using ROS messages. On the Unity side, the ROSsharp library was used to establish communication with the ROSbridge Unity node in the broker using TCP WebSockets. The Unity MixedReality-Toolkit 2 (MRTK2) was used, in addition to some other libraries and APIs.
2. Project Initiation: After finishing the programming phase, the application was ready to run. First, the operator needed to launch the system by linking the real and digital worlds using the immersive MR head-mounted device. Thanks to the depth sensor, IMU motion sensor and integrated camera on the Hololens 2 device, the spatial mapping of the real environment was provided. The link was then made using an initial calibration that enabled a connection between the coordinate frame of the Hololens 2 device and the real frame of the robot. In our use case, the calibration was performed using an external Vuforia SDK [44]. The application was launched once the image target that was placed next to the robot was scanned, as shown in Figure 7.

There were many other possibilities that were tested, such as AI-based object recognition algorithms and QR code scanning, but the image target method from Vuforia was selected because of its simplicity, efficiency and ease of implementation. Once the operator put the Hololens 2 device on and launched the app, the device started the spatial scanning of the real world. As soon as the target was detected, the previously implemented virtual objects appeared in the holographic lenses as though they were real.

3. Model Superimposition: The superimposition of the digital twin of the robot was a critical phase for accurate and efficient simulation results. First, the digital twin had to be overlaid onto its physical twin. By scanning and detecting the Vuforia camera-based image target during the project initiation phase, as shown in Figure 7, the base coordinate frame of the physical robot was linked to the frame of the digital twin. The system computed the 6-DoF pose of each object, so the position of each object was well known in the real environment. The digital twin, which was implemented by Unity3D, had a left-handed coordinate system, which was different from the frame of the right-handed robot; thus, it required rotational transformation.
4. Holographic Visualization and Interaction: The visualization phase enabled the user to interact with the digital content through the immersive holographic interface.

**Figure 8:**
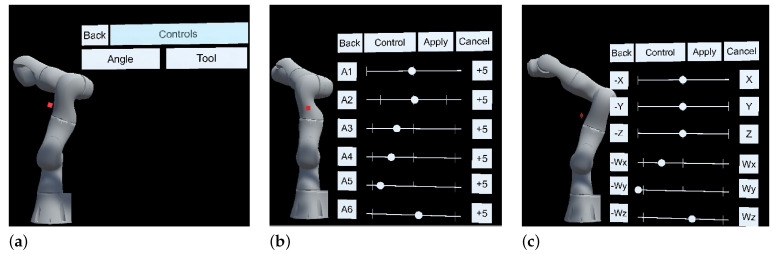
The manual manipulation interaction interfaces: (**a**) main menu panel; (**b**) joint positions jogging type; (**c**) Cartesian coordinate system jogging type.

Interactions were conducted through the user’s hand gestures via the hardware (camera and IMU motion sensor) and software (MRTK2) capabilities of the MR-HMD. Different control panels were implemented, depending on the task, e.g., synchronization, simulation, online programming using manual manipulation or autonomous programming. Figure 8 shows the main panel interface when manual manipulation was chosen. Then, two possible choices were available: “Angle”, which led to the interface presented in Figure 8a and in which forward kinematics was applied, or “Tool”, which led to the interface presented in Figure 8b and in which inverse kinematics was applied.

**Figure 9:**
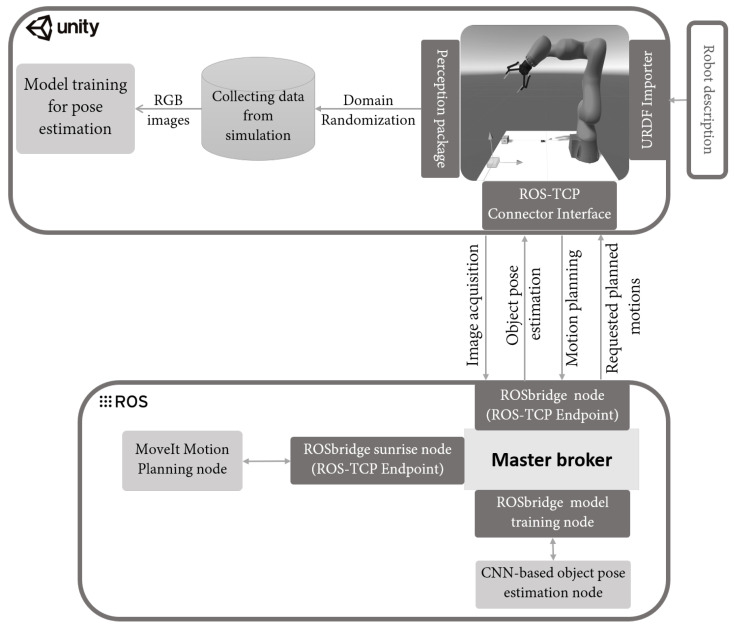
The RoS-based communication procedure during the autonomous decision-making process.

5. Model Training, Object Pose Estimation and Motion Planning: The model training and motion planning nodes aimed to determine the target position and orientation, called pose estimation, as well as the joint position of the robot. This information was calculated using the ROS-based broker, as shown in Figure 9.

The autonomous decision-maker broker, which is presented in Figure 4, comprised an ROS-based system running on a separate computer and had several roles. It was composed of different ROS nodes, one for each service. The motion planning node trained the models to generate the robot trajectory after collecting data, using AI algorithms and the MoveIt Motion Planner [45].

An AI-based algorithm was integrated into the system and used in the use case. The selected algorithm was a deep conventional neural network (DCNN), which was adapted from [46]. It aimed to train the Cartesian position and orientation of the virtual objects during simulation using domain randomization in order to execute simple pick and place tasks. Since simulation was executed in the real world, the environmental parameters, as well as surrounding objects, could change at any time. Thus, the DCNN domain randomization-based method was optimal to cover as many of the attainable scenarios as possible. Two phases were required to implement this method: (1) the collection of a large amount of data from the simulation; (2) the training of the DCNN model.

**Figure 10:**
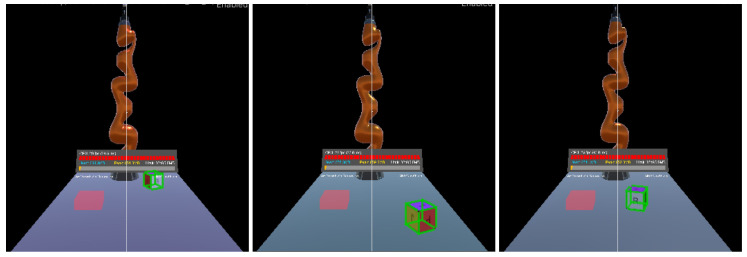
Examples of the stored RGB images that were captured during the simulation.

A major benefit of using the virtual world is the creation of training models in a manageable and personalized way. It also affords automatic data annotation that can be systematically validated. Thanks to its Perception Package, Unity is able to generate large-scale datasets for model training and validation by applying domain randomization, which adjoin ground truth annotations for every captured frame. Randomized parameters provide more variations in the produced data than training a model in a particular rigid environment. The randomized parameters in our use case were cube position and orientation, light direction and light color. The collected data in the use case were RGB images that were taken by the main Unity camera with a specific resolution and 3D bounding boxes, which were labeled scene objects. Thousands of pictures were captured, labeled and stored. Figure 10 presents some examples of the collected images.

**Figure 11:**
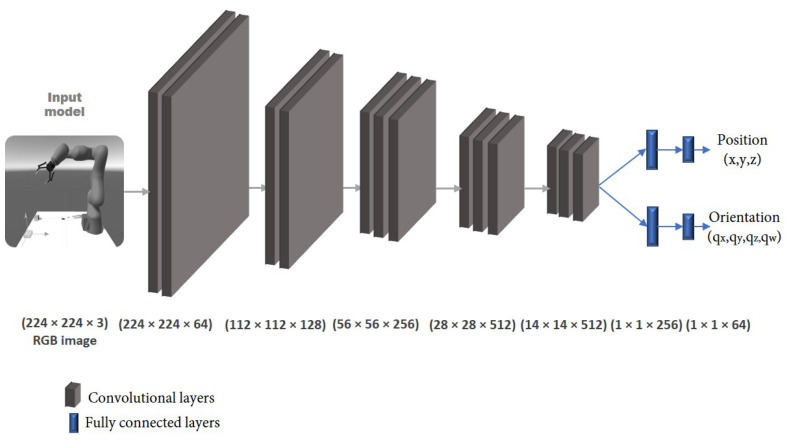
The VGG-16-based CNN architecture of the training model.

Next, the dataset that was gathered from the virtual environment was used to train the DCNN model to find the target position and rotation. The applied model was adapted and modified from [46]. Its architecture is shown in Figure 11, which was based on the convolutional neural network (CNN) VGG-16 architecture. The input models were the previously collected RGB images with a fixed size of 224 × 224. The fully connected layers were downsized to 256 × 64. The network was composed of five sets of convolutional layers. The outputs from the head of the network were (1) (x,y,z) for position and (2) (qx,qy,qz,qw) for orientation.

6. Simulation: The simulation phase is not mandatory, but it is one of the benefits of the proposed method. Users could visualize the planned movements being executed on the virtual robot before they were transmitted to the real robot. Simulation is beneficial while working in hybrid teams. It reduces the defects of the robot and nearby equipment and maintains operator safety. It also permits real-time simulation in real environments, which cannot be achieved through traditional simulation methods that use monitors or VR-based methods.
7. Validation: Once the operator agreed to a simulation, it could be validated by pressing the “Apply Movements” button on the interface. Validation meant that the simulated movements could be transmitted and executed by the real robot in the real world.
8. Outcome Transmission: After the validation, the simulated outcomes were transferred to the broker using ROSbridge, where they were processed and transformed into robotic commands.

## 5. Results and Discussion

The results from the use of the proposed DT-HRI framework in a use case are presented in this section.

The main objective of this study was to apply the proposed DT method to a realistic use case to evaluate its performance. The described system was implemented, deployed using the Hololens 2 device and then demonstrated. The goals of the approach could fail for many reasons, such as the system configuration, real-time communication between the physical and digital worlds, autonomous decision-making prospects or system reconfiguration flexibility. Once deployment was complete, the operator could simulate and program the robot without any previous programming knowledge just by using hand gestures to interact with the robot through the immersive interface.

Communication is a critical point that should be maintained and efficient throughout the system process. During the first Connect step, the digital and physical twins needed to be connected and able to send and receive data in real time. The ROS-based communication interfaces were well established and tested. Once the master was initiated, the robot controller was connected and started its waiting loop for received requests, then it started publishing. In the same way, the digital twin was also configured and connected via the IoT gateway. All connections were made through the TCP/IP connection of the IoT gateway.

**Figure 12:**
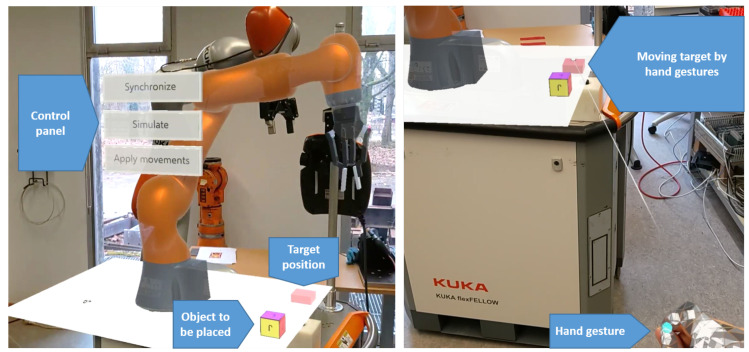
The autonomous simulation of the digital twin through the Hololens 2 device. The combined digital and physical worlds contained the real robot, its digital twin, an immersive user interaction interface control panel, a digital cube object to be placed by the virtual robot and a cube representing the target position where the object was to be placed.

The collection of data from physical twin was performed by clicking the “Synchronize” button, as shown in Figure 12. At this moment, the robot controller started publishing the necessary state. The published values from the robot controller were precise. The data were transmitted in real time to the digital twin through the IoT gateway. After the transformation and calculation, the published values were applied to the digital twin. The digital twin then had the same position and configuration as the physical model. Until this point, the system was running in real time and without errors.

While trying to combine both worlds, the system used first image target recognition to position the digital environment within the real world, so that it overlaid the physical twin. The stability of the visualized holograms after the image target scanning was evaluated and an average displacement error of ±1 to 2 cm was detected between the two bases of the robots while moving around them. Taking into consideration the size of the objects, this error did not impact the pick and place tasks, especially when using the 2-finger gripper. Using advances in hardware capabilities, software APIs and detection algorithms, this error could be reduced.

Once the digital world was set up and obtained its position from the real world, the user could start the interaction and simulation process. Firstly, the user needed to choose the type of operation: either manual or autonomous manipulation. The manual manipulation of the robot involved the user applying either forward or inverse kinematic robot movements through the Hololens UX interface, as shown in [47]. Otherwise, the user could select the autonomous mode via the control panel. Then, a UX control panel appeared through the device’s holographic view, as shown in Figure 12. Next, three choices were possible: (1) synchronize the digital and physical models by sending the current joint positions of the real robot to its digital twin; (2) simulate the motion planning algorithm by moving the target position using hand gestures, followed by the pick and place of the cube object thanks to the CNN-trained model; (3) apply movements by sending the planned motions, including the initial positions and target positions, to the real robot.

To pick and place the cube in the desired position, the user used hand gestures to move the small cube representing the target position to the desired position, as shown in Figure 12.

As for future work, AI-based algorithms, as well as the spatial awareness capability of the Hololens 2 device, will be tested to replace the Vuforia image target for the digital model superimposition to reduce displacement errors. Furthermore, thanks to the flexibility of the DT-HRI concept, more demonstrators will be built using different kinds of robots, such as mobile robots, universal robots, etc. The framework will also be extended to implement more tasks, such as collaborative assembly and disassembly operations, resolving shape puzzles, stacking objects of different shapes, etc.

The proposed DT-HRI demonstrator was tested by people who were trying to program the IIWA robot for the first time and total agreement was received from all participants regarding the ease of interaction with the robot without any previous knowledge or training.

## 6. Conclusions

The introduction of Industry 4.0 technologies has reshaped the old form of manufacturing. Despite the existence of technologies such as IoT, CPS, AI and collaborative and autonomous robots in industrial environments and even though the main objective of Industry 4.0 is to implement better connected, more flexible and smarter industrial environments, some aspects still need to be better integrated and implemented. Among these aspects are human–robot interactions, collaborative robot programming and simulation, which still need many improvements in order to fit in with new smart environments in which robots and humans work together in hybrid teams.

Common CPS, as well as DT systems, are currently used for either offline virtual simulation or for linking digital and physical twins. MR technology is commonly used for visualization tasks, such as enhanced maintenance or remote assistance. IoT communication designs are well integrated and provide real-time communication but, at the same time, are still complex when developing or integrating new devices. The evolution of collaborative robots ought not to exclude humans but to facilitate their work and enhance their efforts when needed.

The proposed work envisioned the future of robot programming and robot simulation in industrial environments in which humans and robots work side by side in hybrid teams. The main objective of this work was to build and demonstrate a new digital twin framework, which was designed to enhance human–robot interactions, robot programming and simulation. The proposed approach would be required to create a flexible real-time service-based framework for both vertical and horizontal integration. It would also be needed to provide intuitive and human-friendly usage for unskilled workers.

This paper introduced the six steps of the digital twin for the human–robot interaction framework, which was adapted and modified from the common 5-C architectural design of CPSs. Its flexible architecture granted the robust integration of new devices, systems and APIs. Since this framework was initially designed for human–robot interactions, its capabilities and implementation were demonstrated through a use case study. In all six steps, a horizontal technological architecture was used.

Even though many hardware, as well as software, problems and challenges were faced during the development and demonstration of the use case, the major contributions of this research are (1) the proposal of a DT concept methodology for HRIs, (2) the application of the proposed method in a specific use case and (3) the implementation of the use case using a selection of hardware equipment, software, APIs and algorithms.

The proposed system aims to (1) facilitate human–robot interactions and robot programming tasks so that they are feasible for any unskilled worker and (2) provide more realistic and efficient simulations in a real environment under real conditions.

## Figures and Tables

**Table 1:** A comparison of different programming methods.

Characteristics	Online	Text-Based Offline	VR-Based Offline
Robot programming by unskilled workers (non-engineers)	Applicable	Not Applicable	Not Applicable
Teach complex tasks	Not Applicable	Applicable	Applicable
Preview movements in a real environment	Not Applicable	Not Applicable	Not Applicable
Efficient and human-friendly interactions	Applicable	Not Applicable	Not Applicable
Easy integration of new devices or software capabilities	Not Applicable	Applicable	Applicable

**Table 2:** Information on the physical equipment, integrated sensors and their outcomes.

Equipment or Device	Sensor	Information
Physical Collaborative Robot	Integrated torque sensors	Joints values
	Attached gripper	Gripper state
MR-HMD	Camera	Real environment mixed with digital content (photos and videos)
	Depth sensor	Spatial mapping of the real-time environment mesh
	Inertial measurement unit (IMU) motion sensor (e.g., accelerometer, gyroscope, magnetometer, etc.)	World-scale position tracking

## Data Availability

Not applicable.
